# Characteristics and Surgical Management of Bilateral Body Mandibular Fractures: A 23-Year Experience

**DOI:** 10.3390/jcm14010160

**Published:** 2024-12-30

**Authors:** Fabio Roccia, Paolo Cena, Giulia Cremona, Paolo Garzino Demo, Federica Sobrero

**Affiliations:** Division of Maxillofacial Surgery, Surgical Science Department, Città della Salute e della Scienza Hospital, University of Turin, 10126 Turin, Italy

**Keywords:** mandibular fractures, osteosynthesis, etiology, facial trauma

## Abstract

**Background/Objectives**: Mandibular fractures are among the most common facial injuries. Bilateral fractures of the mandibular body region (BBMFs), however, are rare. The aim of this retrospective study was to analyze the characteristics, surgical management, and outcomes of BBMFs in a third-level trauma center in northern Italy. **Methods**: Between 1 January 2001 and 31 December 2023, the following data were collected about patients hospitalized for BBMFs: age, sex, cause of fracture, dental status, degree of mandibular atrophy, surgical approach, number and thickness of plates used, concomitant maxillofacial fractures, length of hospital stay, and outcomes. Statistical analysis was performed using SPSS software. **Results**: During the study period, 26 patients (11 males and 15 females) presented with BBMFs, of which five were dentate (median age, 19 years) and 21 edentulous (median age, 80 years). The primary cause of trauma was road traffic accidents (RTAs) in dentate patients and falls in edentulous patients. In most dentate patients, fractures were treated using an intraoral approach with rigid or mixed fixation, using ≤1.4 mm thick plates. Edentulous patients were primarily treated using an extraoral approach and rigid fixation with ≥1.5 mm plates. The use of plates ≥ 1.5 mm was statistically associated with edentulous patients (*p* = 0.042) and with increasing degrees of atrophy (*p* = 0.020). **Conclusions**: This study shows that BBMFs are uncommon injuries, associated with high-impact trauma in dentate patients and medium- or low-impact trauma, such as falls, in edentulous patients. Internal fixation was predominantly rigid, with thicker plates used as the degree of mandibular atrophy increased.

## 1. Introduction

Mandibular fractures are among the most common sites of injury in facial trauma, with an incidence ranging from 20% to 56% [1,2]. They are usually caused by road traffic accidents (RTAs), falls, and violence, and they predominantly affect young adult males [3,4,5,6]. A recent multicenter prospective European study [7] showed a similar incidence of single and double mandibular fractures, primarily localized in the angle region for unilateral fractures and in combination with the angle and parasymphyseal regions for bilateral fractures, consistent with the literature [8,9,10].

The angle, due to its thin cross-sectional area and the presence of the third molars, and the parasymphyseal region, particularly the area of the mental foramen, are well-recognized areas of weakness in the mandible. Therefore, depending on the direction and severity of the impact force, these sites are more frequently fractured, along with the condylar region [11,12,13].

By contrast, mandibular body fractures are less common, with percentages ranging from 9% [14] to 25% [15] of all mandibular injuries and are mostly associated with concomitant angle or condyle fractures [16,17].

The relatively low incidence of these fractures is believed to be due to the fact that when impact is delivered from a lateral source, the dentition and occlusion can absorb the external force transmitted along the body region [11,13,18]. Instead, in edentulous patients, the mandible is prone to severe bone resorption [19,20] and, as reported by Jacobs [21], the posterior mandibular region, particularly the body, is more susceptible to this phenomenon due to its less dense bone compared to the anterior part. Therefore, the atrophic mandible has a specific area of weakness in the body region, and even mild trauma can lead to fractures at this site.

Several authors reported on the treatment of mandibular body fractures, either isolated or associated with other mandibular fractures, but to our knowledge, in the literature, there is scanty evidence about the surgical management of bilateral body mandibular fractures (BBMFs), with most of the papers being focused on edentulous atrophic mandibles [4,5,22,23,24]. In fact, in the dentate population, BBMFs are an unusual traumatic event: Moura et al. [9] found only three patients with BBMFs in a 7-year retrospective analysis, while Buch et al. [25] found six patients over two years and a half. On the other hand, in edentulous patients, BBMFs are relatively more frequent: Ellis and Price [26] studied 32 atrophic mandibular fractures and reported that 26 were bilateral, most of them in the body region. In a retrospective analysis of 55 patients with atrophic mandibular fractures, Gerbino et al. [27] reported 22 unilateral fractures and 23 bilateral body fractures with percentages similar to those reported by Eyrich et al. [28].

Unlike mandibular fractures in dentate patients, the surgical management of atrophic mandibular fractures still represents a challenge due to poor bone stock, reduced osteogenesis, and decreased blood flow [26,27,29].

The aim of this 23-year retrospective study was to analyze the characteristics, surgical management, and outcomes of BBMFs in a regional third-level trauma center in northern Italy.

## 2. Materials and Methods

This retrospective study included patients with BBMFs hospitalized in the division of Maxillofacial Surgery at Città della Salute e della Scienza Hospital, Turin, Italy, between 1 January 2001 and 31 December 2023. Data were retrieved from a dedicated database updated daily on all patients admitted for maxillofacial fractures.

Collected data included age, sex, cause of fracture (fall, RTA, assault, sports- and work-related injuries), dental status (dentate or edentulous), degree of mandibular atrophy (according to Luhr, Reidick, and Merten classification [30]), surgical approach (intraoral, extraoral, combined intraoral and extraoral, translesional), number and thickness (≤1.4 mm or ≥1.5 mm; locking/non-locking system) of plates used for open reduction and internal fixation (ORIF), concomitant maxillofacial fractures, length of hospital stay, and outcomes. Minimum follow-up was set at 6 months.

Osteosynthesis was defined as “non-rigid” when the fracture was fixed with a single plate ≤ 1.4 mm thick, “rigid” when a single plate ≥ 1.5 mm in thickness or at least two plates of any thickness were employed, and “mixed” when one fracture was rigidly fixed and the other was non-rigidly fixed [10,29,31,32,33].

### Statistical Analysis

Statistical analysis was performed using SPSS software (version 28.0.1.0, IBM Corp., Armonk, NY, USA). The association between age and hospital stay with dental status was analyzed using the Mann–Whitney U test, given the non-normal distribution of the variables. The association between the cause of fracture, surgical approach, and type of treatment with dental status was analyzed with Fisher’s exact test, with Bonferroni correction for multiple comparisons. A chi-squared test for trends was applied to analyze the use of ≤1.4 mm or ≥1.5mm plates among different levels of mandibular bone atrophy. All statistical analyses were two-tailed, and the significance level was set at *p* < 0.05.

## 3. Results

During the study period, 1419 patients with mandibular fractures were hospitalized; 235 of them (16.6%) had body mandibular fractures. Among these, 26 patients, of whom five were dentate and 21 edentulous, reported a BBMF.

The dentate patient group consisted of four males and one female, aged between 18 and 56 years (median 19.0 years, IQR [interquartile range] 26.0) (Table 1). BBMF was caused by RTA in three cases and by a work accident and a sports injury in one case each. Two patients had associated middle third fractures.

The fractures were osteosynthesized via an intraoral approach with rigid fixation in four patients, mixed in one patient, almost always using non-locking plates of thickness ≤ 1.4 mm, as summarized in Table 1. The median hospitalization was 5 days (IQR, 3), and the postoperative follow-up was uneventful for all patients.

The edentulous patient group consisted of 14 females and seven males, aged between 37 and 94 years (median 80.0 years, IQR, 26.0) (Table 2). The main cause of fracture was fall (15 patients), followed by RTAs (3), work-related injuries (2), and assault (1). Among the 12 patients with type III atrophy, 11 patients underwent, via extraoral approach, rigid fixation with ≥1.5 mm locking plates; only one patient received a mixed fixation. Four patients with type II atrophy were rigidly fixed via an extraoral approach. Finally, among the five patients with type I atrophy, rigid fixation was used in three patients and mixed fixation in two, with a prevalence of intraoral approach. Bone grafts were not used in any patient. The median hospitalization was 6 days (IQR, 3) (Table 2).

A transient functional deficit of the marginalis mandibulae branch of the facial nerve was observed in the immediate postoperative period in two patients, where an extraoral approach was performed. Nerve function had fully returned in both patients by the 6-month follow-up visit.

**Figure 1:**
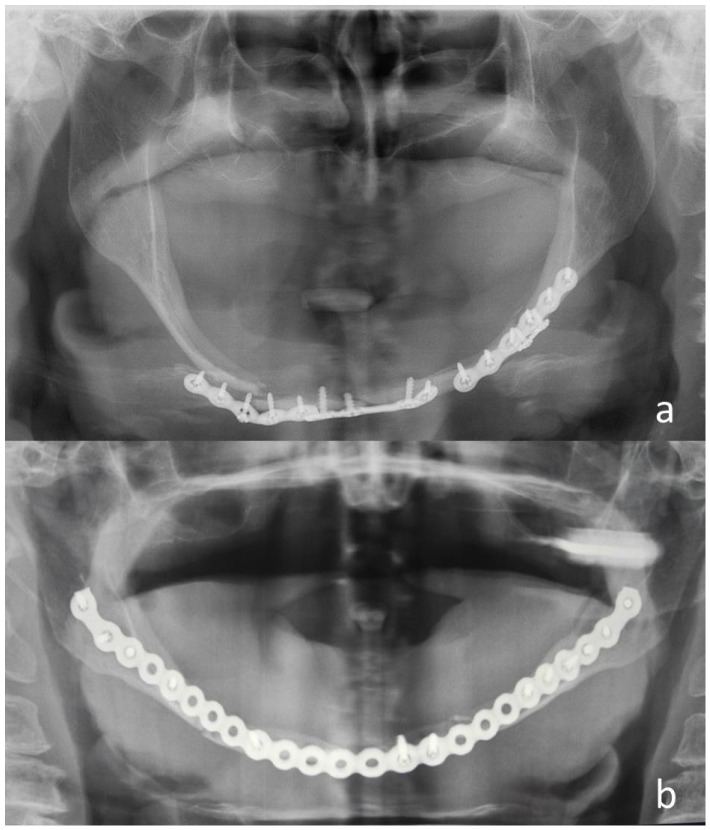
(**a**) Panoramic radiograph showing inadequate fixation in patient No. 18 with Stage III atrophy; (**b**) postoperative panoramic radiograph showing ORIF with a 2.0 mm titanium locking plate.

One patient (No. 18), whose BBMF had been fixated with a 1.5 mm plate at each fracture site, was reoperated two weeks after the first surgery. As shown in Figure 1, one of the plates had dislocated and therefore, both non-locking 1.5 mm plates were replaced with a single 2.0 mm locking plate.

There were no cases of nonunion, infections, wound dehiscence, or loss of fixation due to hardware failure (plate fracture or loss of screws).

Table 3 summarizes the comparison between the two groups. Edentulous patients had a significantly higher median age compared to dentate patients (*p* < 0.001, Mann–Whitney U test).

The causes of fracture were differently distributed between the two groups, and falls were significantly associated with edentulous patients (*p* = 0.008, Fisher’s exact test). The median hospital stay was not significantly different between the two groups (*p* = 0.613 and 0.801, respectively, Mann–Whitney U test). Edentulous patients were treated more frequently using the extraoral approach (86%) compared to dentate patients (0%) (*p* < 0.001, Fisher’s exact test).The use of plates ≥1.5 mm was statistically associated with edentulous patients (57%) compared to dentate patients (0%) (*p* = 0.042, Fisher’s exact test) and, specifically, with increasing degrees of atrophy (20% in atrophy I, 25% in atrophy II, 83% in atrophy III) (*p* = 0.020, chi-squared for trends).

## 4. Discussion

Although mandibular fractures are among the most common injuries encountered by maxillofacial surgeons in trauma care centers, BBMFs are rare in dentate patients, as highlighted in this 23-year retrospective study [3,34]. In edentulous patients, mandibular fractures have a low incidence, ranging from 1% to 5% [35], and the body region is the most common site of injury [36].

The dental status of the mandible resulted in significant differences in both the epidemiological patterns and surgical management between the two groups in this study. BBMFs in dentate mandibles occurred predominantly in young males and were associated with high-impact trauma, particularly RTAs. In contrast, consistent with the previous literature [26,27,28], BBMFs in edentulous mandibles occurred in patients with a significantly higher median age compared to dentate patients (80 years vs. 19 years). Moreover, this type of injury primarily affected females and was caused by falls, which are typical epidemiological characteristics of cranio-maxillofacial trauma in the elderly population [6,37,38].

In the surgical management of BBMFs, ORIF was employed in both dentate and edentulous patients to stabilize the mandible and restore its form and function. In dentate patients, an additional goal was to restore pre-trauma occlusion. However, the methods of internal fixation used differed between the two groups.

When a fracture occurs and it is decided to reduce and stabilize it, it must be considered that the bone and the means of osteosynthesis used for its containment define a complex interactive system [12]. In fact, the stability of the osteosynthesis does not only depend on the thickness and length of the plate as well as on the screws used but also on their positioning, the properties of the material, the methods of application, and the condition of the bone (size, density, cellular orientation). In favorable circumstances and with the application of appropriate osteosynthesis devices, the bone acts as a buttress and provides a guide along which the functional forces act on both sides of the fracture, maintaining a balance of forces and resistance to functional loads. In this way, stabilization sufficient to allow the function without affecting bone healing is recreated. On the contrary, in the atrophic mandible, it is necessary to increase the stability of an osteosynthesis, making it more rigid in order to support the physiological functional loads.

When a fracture occurs, it alters not only the mechanical continuity of the bone but also its biology. The conditions that alter the blood supply to the bone following a fracture largely influence the subsequent healing process, as do the ways in which the circulation is altered, which have a major impact on the outcome. If the vessels supplying the bone are injured or if the trauma that causes the fracture includes major vessels such as a feeding artery, large areas of the bone will be compromised. In all cases, cortical vessels, such as the Haversian and Volkmann canals, are interrupted along the fracture line, and since the intracortical circulation is a low-pressure system, a clot forms within the injured vessels, which stops the bleeding. The interruption of blood flow, in turn, leads to congestion, followed by further clot formation, leaving the edges of the fragments deprived of appropriate blood perfusion. If circulation does not resume within a few hours, the occlusion of the vessels becomes irreversible and the osteocytes in the bone will undergo necrosis. Therefore, good tissue perfusion is a prerequisite for an adequate healing process.

While healing processes occur relatively quickly in soft tissues, the situation is more complex within the bone, especially in compact bone, since a space for new vessels must first be re-canalized, which usually occurs after 2–3 weeks. Areas of necrotic bone are removed by osteoclast activity, starting from the perfused bone and gradually penetrating the necrotic area along re-canalized vascular channels. Newly formed vessels follow the osteoclast activity and at the same time, the osteoblasts rebuild new channels with the addition of bone. Usually, this remodeling is limited to the area where circulation has been compromised, and it is in this “transitional” phase that the healing bone, especially along the fracture edges, appears less radiopaque on X-rays [39].

Surgical treatment may also add to the damage caused by the initial trauma in the blood supply to the bone. In fact, reduction maneuvers, aimed at aligning the fracture stumps, milling for the positioning of screws, and the fixation of plates for osteosynthesis, can further damage the cortical circulation and the blood supply from the periosteal side. However, surgical internal fixation using plates is universally recognized as allowing for a more rapid restoration of the intramedullary circulation and a direct approach to the capillaries from one edge to the other of the bone stumps, factors that, associated with an adequate blood supply and the presence of specific tissue cells, are the prerequisites for a favorable healing of the fracture. Bone healing is defined as primary when absolute immobilization or rigid fixation is established, aimed at preventing inter-fragmentary movements and secondary if a non-rigid fixation is chosen, i.e., not able to prevent such movements [28].

In 1895, Sir William Arbuthnot Lane [40], an English surgeon, performed the first stabilization on displaced orthopedic fractures using rigid plates, as he believed that physiological healing of the bone was only possible when the injury was treated using rigid fixation. Numerous osteosynthesis techniques were therefore developed, mainly based on inter-fragmentary compression, using screws and plates designed to produce axial compression between the bony stumps.

The model of primary bone healing using rigid fixation was then adopted at the end of the 1960s by Luhr [41] and Spiessl [42] for the osteosynthesis of mandibular fractures using rigid plates with dynamic eccentric compression and bicortical screws. Rigid fixation allows for early mobilization even under functional loads, making an intermaxillary block no longer necessary in the post-operative period [43]. For comminuted fractures or with severe bone atrophy, locking plates or reconstruction plates are used to obtain rigid fixation.

Locking plates are designed with threaded holes through which the screw has two separate fixation points, one in the bone and one on the plate. The screws are fixed to the plate independently of the bone and therefore, the plate provides stability to the fracture without requiring direct contact with the bone. For this reason, it is not necessary to adapt the plate to the bone, periosteal damage is limited, as the bone is not compressed by the plate, and finally, complications caused by screw loss are reduced.

Reconstruction plates with a thickness of ≥2.0 mm, on the other hand, require perfect adaptation to the bone to prevent dislocations of the fracture segments and create pressure on the bone when the screws are tightened.

Osteosynthesis plates that provide support and stability to the fracture segments during function, supporting all the functional loads transmitted to the bone, are defined as load bearing.

In 1978, Bruce McKibbin [44], an American orthopedic surgeon, developed the principle of secondary healing in the treatment of fractures in long bones, following the path traced by Julius Wolff [45], a German anatomist and surgeon, who in 1870 developed the theory that relates the bone structure and the mechanical forces exerted on it so that where pressure and tension loads occur on the bone, the formation of a bone callus and its subsequent remodeling occurs. This process is made possible by a non-rigid fixation, which allows for inter-fragmentary micromovements. In the same period, Michelet et al. [46,47] and Champy [48] developed miniplates and monocortical screws for maxillofacial traumatology, which were initially used for the non-rigid fixation of mandibular fractures but later also for the treatment of fractures of the middle third of the facial skeleton. The reduced dimensions of these plates, especially in terms of thickness ≤1.4 mm, offer various advantages such as limited incisions with less dissection and detachment of the soft tissues and periosteum. Furthermore, the monocortical screws allow the plates to be positioned in mandibular areas adjacent to the dental roots, reducing the risk of damaging them. On the other hand, due to the limited thickness compared to the previously described plates, these do not have the same rigidity, and their use requires that the bone stumps participate in the transfer of the functional load along the fractured area. This second condition is commonly defined as load sharing. Non-rigid fixation in the treatment of mandibular fractures with plates is made possible based on the theory of neutralization of mandibular bending and torsion forces in “ideal osteosynthesis lines”. As described by Champy [48], bending forces increase progressively from the symphyseal region toward the ascending branches, while torsional forces act mainly in the symphyseal region, and only these need to be adequately neutralized, suggesting, for example, to use a single miniplate at the upper edge in a subapical position in the body region or along the external oblique line in the mandibular angle region and two in the interforaminal symphyseal region.

Mixed fixation is performed when, in a double mandibular fracture, rigid fixation is used in one location and non-rigid in the other.

Therefore, in all dentate patients, an intraoral approach was used for rigid or mixed ORIF, following the protocols of the Association for the Study of Internal Fixation (AO Foundation) [29] and the Texas school [8,31,32], which recommend a rigid fixation of at least one of the two fracture sites in bilateral mandibular fractures. Although the sample size is small, this study observed a slight trend toward the rigid fixation of both sites, consistent with findings by Singleton et al. [49] in a 30-year monocentric study and Sobrero et al. [7] in a multicenter prospective study on current osteosynthesis strategies for mandibular fractures. This approach reflects over 20 years of accumulated experience in our division, enhancing the ability to adapt and fix plates with a thickness of ≥1.5 mm, thereby minimizing postoperative occlusal discrepancies, as noted by Kearns et al. [50].

In edentulous patients, the type of fixation was influenced by the degree of mandibular atrophy, specifically the vertical height of the bone at the fracture site. Consistent with previous studies [26,27,28,51,52,53,54,55,56] and supported by a recent systematic review [36], the greater the mandibular atrophy, the more rigid the osteosynthesis required, in line with Schilli’s assertion: “The smaller the mandible, the bigger the bone plate must be” [57]. This retrospective study statistically demonstrated the association between increasing degrees of atrophy and the use of plates with a thickness of ≥1.5 mm, most commonly a single locking plate with a thickness of 2.0 mm. As noted by Sikes [58], in cases where the bone height at the fracture site is less than 10 mm, the atrophic mandible does not share of the occlusal load and therefore, the load is beared on the locking plate. According to these considerations, almost all patients (19 out of 21) underwent rigid fixation, with more than half receiving a single plate of 2.0 mm thickness. In line with various authors [26,27,51,59,60], in patients with grades II and III atrophy, ORIF was mainly performed using an extraoral submandibular approach. This method provided adequate exposure of the fractures while preserving the marginal mandibular branch of the facial nerve and the inferior alveolar nerve, which often lies on top of the alveolar crest, as well as the superior and lingual periosteal attachments. According to the surgical technique described in a previous study from our division [27], the bone fragments were reduced and temporarily fixed with a miniplate at each fracture site along the inferior border of the mandible. Subsequently, a single plate ≥1.5 mm in thickness was applied to the lateral side of the mandible and secured with at least three locking screws per side, positioned bilaterally in the angular region and the symphyseal area, where an increased bone stock is available [29,36,59].

In the first decade of our surgical experience, some patients received either a single plate ≥1.5 mm or two plates ≤1.4 mm at each fracture site. However, as exemplified in one case in the present study, the edentulous mandible is subject to various dislocating forces in the body region [36]. In addition, as noted by Ellis and Price [26], miniplates do not provide adequate resistance to the tensile forces generated in the edentulous mandibular body despite the lower masticatory forces compared to dentate patients. Therefore, as recommended by Müller [60] et al. and Gerbino et al. [27], load-bearing osteosynthesis is advised in atrophic mandibular fractures, as rigidity is the most critical factor for successful fracture healing.

The limitations of this study include its retrospective design and the limited number of BBMF cases, particularly among dentate patients. Future multicenter prospective studies with larger cohorts are necessary to further evaluate and validate different surgical management strategies for both dentate and edentulous patients. Larger study samples will hopefully also allow us to take into consideration potential confounders such as age and comorbidities.

## 5. Conclusions

This 23-year retrospective study showed that BBMFs in dentate patients are rare and typically result from high-impact trauma in young males. In contrast, such fractures in edentulous mandibles are relatively more common and occur due to medium- or low-impact trauma in elderly patients. Dental status played a significant role in determining the surgical approach. In dentate patients, an intraoral approach with plates ≤1.4 mm was predominantly used, while the extraoral approach with plates ≥1.5 mm was preferentially used in edentulous patients. In the latter group, an association was also observed between the use of plates ≥1.5 mm and increasing degrees of mandibular atrophy

## Figures and Tables

**Table 1:** Overview of dentate patients with BBMFs enrolled in the present study.

N	Sex	Age	Etiology	Mandibular Fracture Sites Associated	Approach	Treatment	Hospitalization (Days)
1	M	18	Sport	None	Intraoral	2 plates 1.0 mm bilateral	4
2	F	19	RTA	None	Intraoral	1 plate 1.0 mm right 2 plates 1.0 mm left	5
3	M	19	RTA	None	Intraoral	2 plates 1.0 mm bilateral	5
4	M	33	RTA	None	Intraoral	1 plate 1.5 mm left 2 plates 1.0 mm right	9
5	M	56	Work	None	Intraoral	2 plates 1.0 mm bilateral	21

**Table 2:** Overview of edentulous patients with BBMFs enrolled in the present study.

N	Sex	Age	Etiology	Mandibular Fracture Sites Associated	Atrophy Degree *	Approach	Treatment	Hospitalization (Days)
6	M	37	RTA	None	I	Extraoral	1 plate 1.5 mm right 1 plate 1.0 mm left	5
7	F	49	Fall	None	I	Extraoral	Plate 2.0 mm	9
8	M	55	Assault	Symphysis	I	Intraoral	2 plates 1.0 mm left 1 plate 1.0 mm right	3
9	M	55	Work	None	I	Intraoral	1 plate 1.5 mm bilateral	6
10	M	60	Work	None	I	Intraoral	1 plate 1.5 mm bilateral	3
11	M	56	Fall	None	II	Extraoral	2 plates 1.0 mm bilateral	4
12	F	76	Fall	None	II	Extraoral	Plate 2.0 mm	7
13	M	77	Fall	None	II	Extraoral	2 plates 1.0 mm bilateral	6
14	F	81	RTA	None	II	Exraoral	Plate 2.0 mm	8
15	F	76	Fall	None	III	Extraoral	2 plates 1.0 mm bilateral	3
16	F	80	Fall	None	III	Extraoral	Plate 2.0 mm	5
17	F	80	Fall	None	III	Extraoral	1 plate 1.5 mm right 1 plate 1.0 mm left	5
18	F	81	Fall	Symphysis	III	Extraoral	1 plate 1.5 mm bilateral	6
19	F	81	Fall	None	III	Extraoral	Plate 2.0 mm	11
20	F	82	Fall	Symphysis	III	Extraoral	Plate 2.0 mm	4
21	F	84	Fall	None	III	Extraoral	Plate 2.0 mm	6
22	F	84	Fall	None	III	Extraoral	Plate 2.0 mm	8
23	F	85	Fall	None	III	Extraoral	Plate 2.0 mm	7
24	F	87	Fall	None	III	Extraoral	Plate 2.0 mm	7
25	M	90	RTA	None	III	Extraoral	Plate 2.0 mm	4
26	F	94	Fall	None	III	Extraoral	Plate 2.0 mm	7

* Luhr, Reidick, and Merten classification.

**Table 3:** Comparison between dentante and edentulous patients with BBMFs.

	Dentate Patients *n*	Edentulous Patients *n*	*p* Value
**Age** (years)			
median (IQR)	19.0 (26.0)	80.0 (26.0)	<0.001 *
**Cause**			
Assault	0	1 (5%)	0.008 °
Fall	0	15 (71%)	
Work	1 (20%)	2 (10%)	
RTA	3 (60%)	3 (14%)	
Sport	1 (20%)	0	
**Hospital stay** (days)			
median (IQR)	5 (3)	6 (3)	0.801 *
**Surgical approach**			
Intraoral	5 (100%)	3 (14%)	<0.001 °
Extraoral	0	18 (86%)	
**Type of treatment**			
Plates 2.0 or 2.5mm	0	12 (57%)	0.042 °
Plates 1.0 or 1.5 mm	5	9 (43%)	

* Mann–Whitney U test; ° Fisher’s exact test.

## Data Availability

Data is contained with the article.
